# 2-Amino-5-oxo-4-(thio­phen-2-yl)-5,6,7,8-tetra­hydro-4*H*-chromene-3-carbo­nitrile

**DOI:** 10.1107/S2414314624010496

**Published:** 2024-11-08

**Authors:** Carren Nyapola, Sizwe J. Zamisa, Eric M. Njogu, Bernard Omondi

**Affiliations:** a School of Chemistry and Physics, University of KwaZulu-Natal, Westville campus, Private bag X54001, Durban, 4000, South Africa; b Multimedia University of Kenya, PO Box 15653-00503, Nairobi, Kenya; Benemérita Universidad Autónoma de Puebla, México

**Keywords:** crystal structure, pyran, chromenone, hydrogen bond

## Abstract

The crystal structure of the title compound, C_14_H_12_N_2_O_2_S, reveals two symmetrically independent mol­ecules within the asymmetric unit. Each mol­ecule contains a chromenone core attached to a 2-thio­phene ring, cyano, and amino groups. The 2-thio­phene ring of one of the two mol­ecules in the asymmetric unit was found to be disordered over two positions, with the major component having a site occupancy factor of 0.837 (2). The 2-thio­phene ring is nearly orthogonal to the fused 4*H*-pyran ring, with dihedral angles between the two sets of planes being 89.5 (5) and 89.63 (8)°. Inter­molecular hydrogen bonding, involving N—H⋯N and N—H⋯O inter­actions, creates two distinct motifs leading to the formation of a two-dimensional supra­molecular network along the crystallographic *ac* plane.

## Abstract 2

In the crystal structure of the title compound, inter­molecular N—H⋯N and N—H⋯O hydrogen bonds form a two-dimensional supra­molecular network along the *ac* plane, contributing to the cohesion of the crystal.

## Graphical abstract

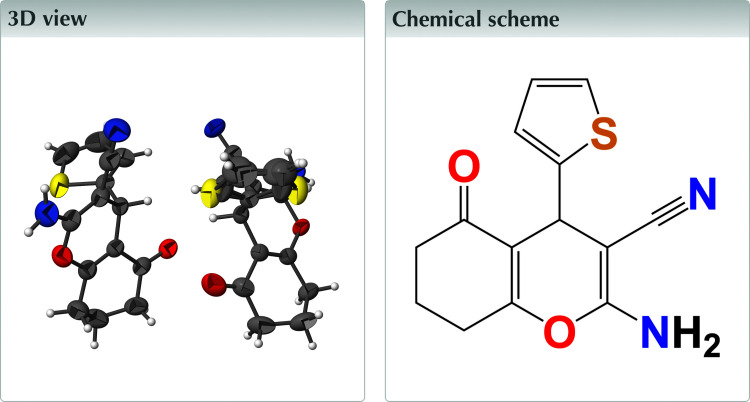

## Structure description

Pyran-based 2-amino-3-carbo­nitrile derivatives are known to possess inter­esting pharmacological properties, which enable them to be explored as potential anti­cancer (Braga *et al.*, 2022 ▸), anti­oxidant (Symeonidis *et al.*, 2009 ▸), anti­bacterial (Kathrotiya & Patel, 2012 ▸), anti­fungal (Alvey *et al.*, 2009 ▸), hypotensive (Cai *et al.*, 2009 ▸) and anti­leishmanial (Narender *et al.*, 2004 ▸) agents. In our previous work, we surveyed the Cambridge Structural Database (CSD) and categorized the diverse inter­molecular hydrogen-bonding patterns that emerge in the crystal structures of aryl-based *rac*-2-amino-3-carbo­nitrile derivatives (Zamisa *et al.*, 2022 ▸). The study revealed that the propensity of inter­molecular hydrogen bonds that involve the compounds amino functional group could be linked to their binding affinity towards *calf thymus* de­oxy­ribonucleic acid, which aligned with our quest for developing potential anti­cancer agents (Adeleke *et al.*, 2022 ▸, 2023 ▸; Zamisa *et al.*, 2023 ▸).

**Figure 1:**
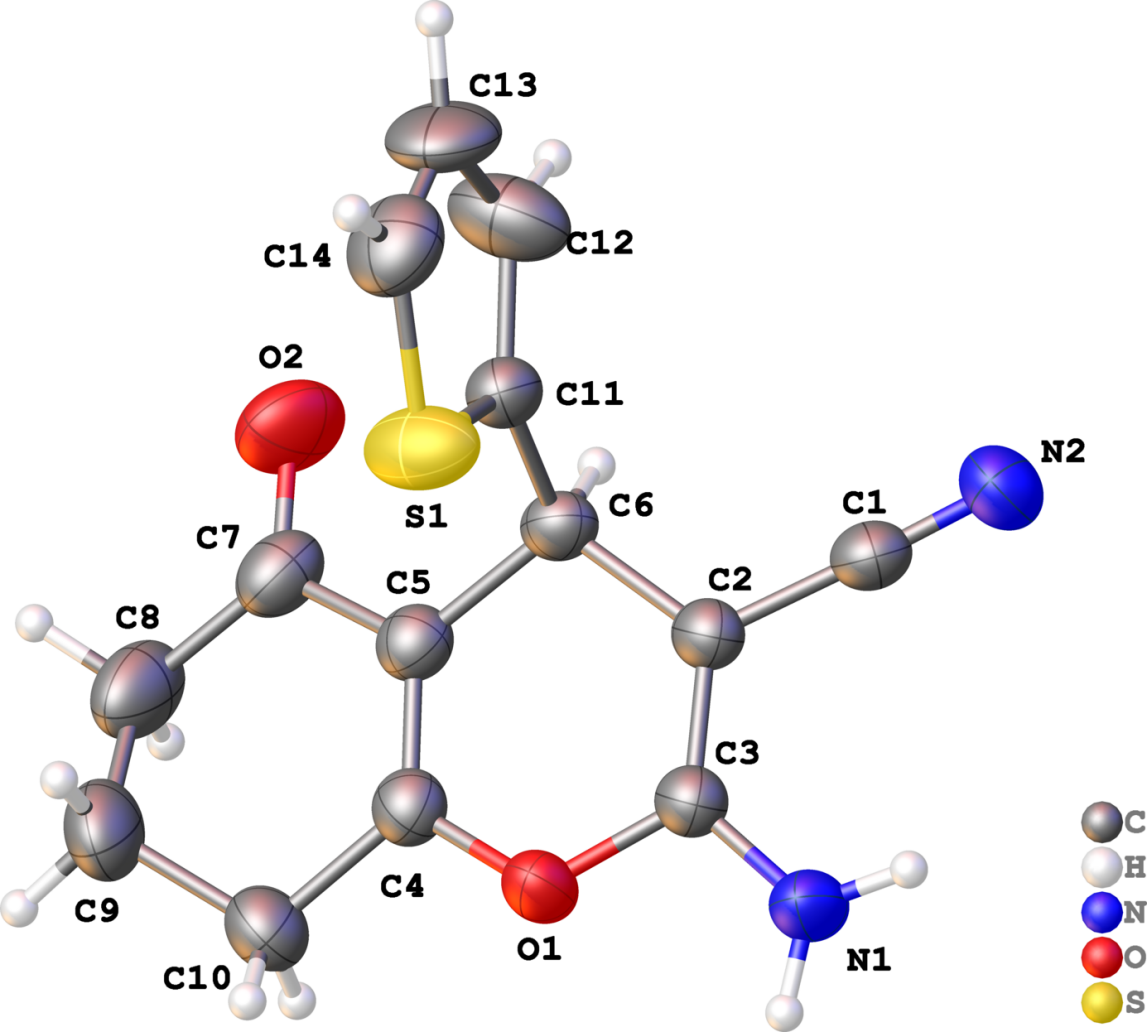
Mol­ecular structure of the title compound with displacement ellipsoids drawn at the 50% probability level. Only one of the two mol­ecules in the asymmetric unit is shown for clarity.

**Figure 2:**
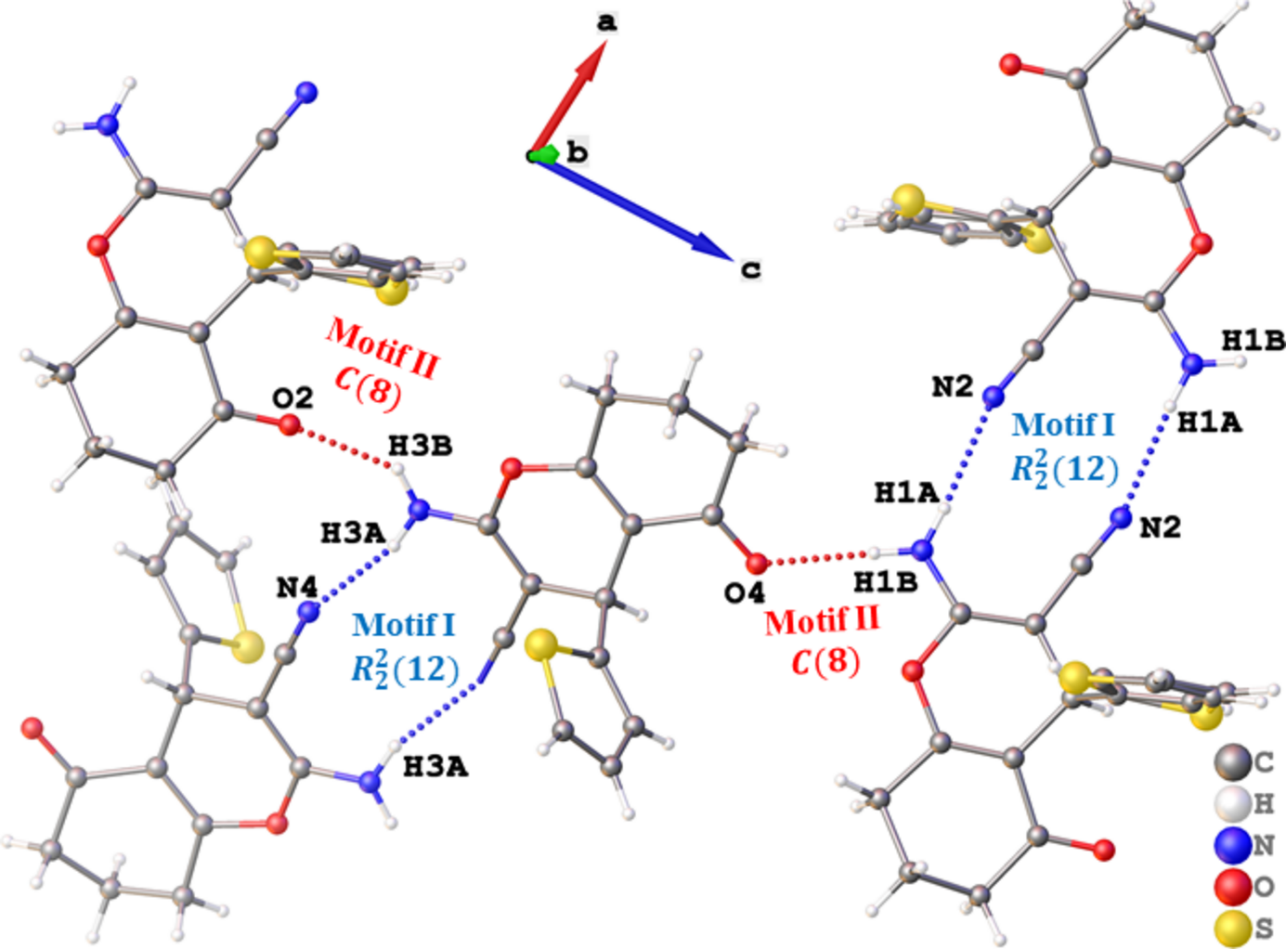
Representation of inter­molecular N—H⋯N (blue dotted bonds) and N—H⋯O (red dotted bonds) hydrogen-bonding patterns which are categorized as motifs **I** and **II**, respectively.

**Figure 3:**
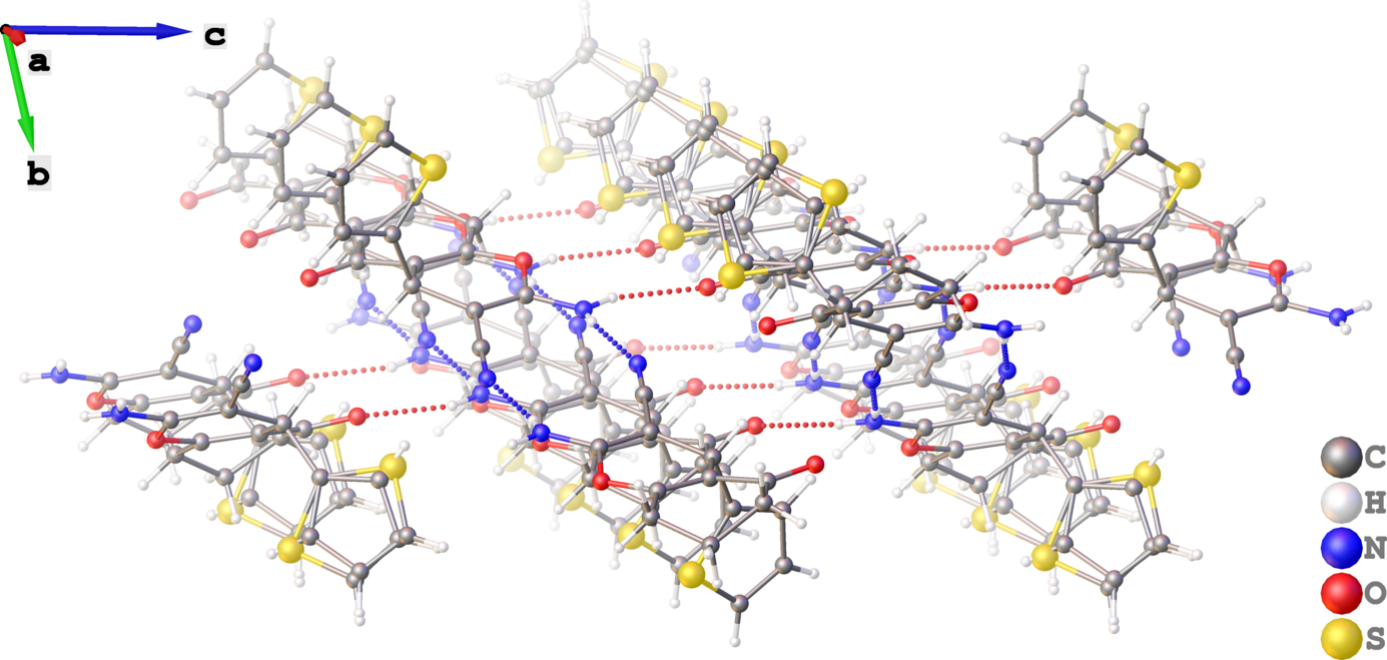
Representation of the two-dimensional supra­molecular architecture that results from inter­molecular N—H⋯N and N—H⋯O hydrogen bonds.

The crystal structure of the title compound consists of two symmetrically independent mol­ecules in the asymmetric unit. Each mol­ecule consists of a chromenone moiety with a 2-thio­phene, cyano and amino groups attached to it, as shown in Fig. 1 ▸. The 2-thio­phene rings are orthogonal to the fused 4*H*-pyran rings, with measured dihedral angles of 89.5 (5) and 89.63 (8)°, which is slightly wider than that of 2-amino-7,7-dimethyl-5-oxo-4-(thio­phen-2-yl)-5,6,7,8-tetra­hydro-4*H*-1-ben­zo­pyran-3-carbo­nitrile [85.80 (8)°; Zamisa *et al.*, 2022 ▸]. In the crystal packing of the title compound, inter­molecular hydrogen-bonding patterns engendered by the amino functional group were found (Fig. 2 ▸). One of amino functional group’s hydrogen atoms (H1*A* or H3*A*) inter­acts with the nitro­gen atom (N2 or N4) of the cyano group in a neighbouring mol­ecule, *via* N—H⋯N hydrogen bonds (Table 1 ▸), with graph-set descriptor 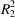
(12). The other hydrogen atom of the amino functional group (H1*B* or H3*B*) forms N—H⋯O hydrogen bonds [graph-set descriptor *C*(8)] with the carbonyl oxygen atom (O4 or O2) of a neighbouring mol­ecule (Table 1 ▸). The inter­molecular N—H⋯N and N—H⋯O hydrogen bonding patterns are categorized as motif **I** and **II**, respectively, according to the literature (Zamisa *et al.*, 2022 ▸). The combination of the two hydrogen-bonding motifs results in the formation of a two-dimensional supra­molecular structure that extends along the crystallographic *ac* plane as shown in Fig. 3 ▸.

## Synthesis and crystallization

A mixture of malono­nitrile (0.01515 mmol), 2-thio­phene­carboxaldehyde (0.01515 mmol), 1,3-cyclo­hexa­nedione (0.01515 mmol), and two drops of tri­ethyl­amine added as a catalyst, was poured in a 35 ml microwave reaction vessel, which was closed using a snap-on cap. The same microwave radiation-assisted synthesis protocol described in the literature was followed (Zamisa *et al.*, 2022 ▸). This reaction was closely monitored by thin-layer chromatography with a solvent mixture of ethyl acetate:hexane (1:1 ratio, *v*/*v*). A brown solid mass precipitated from the reaction mixture and was filtered off *in vacuo*. The crude product was then purified by recrystallization from an ethano­lic solution, to give brown block-shaped crystals.

## Refinement

Crystallographic data and structure refinement details are summarized in Table 2 ▸. The 2-thio­phene ring of one of the two mol­ecules of the title compound in the asymmetric unit was found to be disordered over two positions. PART instructions were used to model the disorder (Sheldrick, 2015*b* ▸), with the major component having a site occupancy factor of 0.837 (2). Furthermore, the refinement of the disordered 2-thio­phene ring was kept stable with SADI, SIMU and DELU restraints in *SHELXL*. The displacement parameters of the equivalent atoms of the disordered components were kept the same using an EADP instruction (Sheldrick, 2015*b* ▸). Finally, the hydrogen atoms of the amino functional group were located and assigned from a difference map, but the N—H bond lengths were restrained to 0.860 (1) Å using *DFIX* instructions.

## Supplementary Material

Crystal structure: contains datablock(s) I. DOI: 10.1107/S2414314624010496/bh4090sup1.cif

Structure factors: contains datablock(s) I. DOI: 10.1107/S2414314624010496/bh4090Isup2.hkl

Supporting information file. DOI: 10.1107/S2414314624010496/bh4090Isup3.cml

CCDC reference: 2394700

Additional supporting information:  crystallographic information; 3D view; checkCIF report

## Figures and Tables

**Table 1:** Hydrogen-bond geometry (Å, °)

*D*—H⋯*A*	*D*—H	H⋯*A*	*D*⋯*A*	*D*—H⋯*A*
N3—H3*A*⋯N4^i^	0.86 (1)	2.25 (1)	3.089 (3)	164 (2)
N3—H3*B*⋯O2^ii^	0.86 (1)	2.10 (2)	2.794 (2)	138 (2)
N1—H1*A*⋯N2^iii^	0.86 (1)	2.25 (1)	3.105 (2)	174 (2)
N1—H1*B*⋯O4^iv^	0.86 (1)	2.13 (1)	2.984 (2)	176 (2)

Symmetry codes: (i) 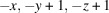; (ii) 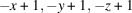; (iii) 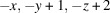; (iv) 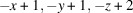.

**Table 2:** Experimental details

Crystal data	
Chemical formula	C_14_H_12_N_2_O_2_S
*M*_r_	272.32
Crystal system, space group	Triclinic, *P* 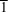
Temperature (K)	296
*a*, *b*, *c* (Å)	8.6822 (2), 10.0623 (2), 16.5504 (4)
α, β, γ (°)	77.201 (1), 84.053 (1), 73.839 (1)
*V* (Å^3^)	1352.89 (5)
*Z*	4
Radiation type	Mo *K*α
μ (mm^−1^)	0.24
Crystal size (mm)	0.22 × 0.18 × 0.13

Data collection	
Diffractometer	Bruker APEXII CCD
Absorption correction	Multi-scan (*SADABS*; Krause *et al.*, 2015 ▸)
*T*_min_, *T*_max_	0.710, 0.746
No. of measured, independent and observed [*I* > 2σ(*I*)] reflections	31629, 5315, 3991
*R*_int_	0.030
(sin θ/λ)_max_ (Å^−1^)	0.617

Refinement	
*R*[*F*^2^ > 2σ(*F*^2^)], *wR*(*F*^2^), *S*	0.044, 0.127, 1.05
No. of reflections	5315
No. of parameters	368
No. of restraints	14
H-atom treatment	H atoms treated by a mixture of independent and constrained refinement
Δρ_max_, Δρ_min_ (e Å^−3^)	0.36, −0.37

Computer programs: *APEX2* and *SAINT* (Bruker, 2009 ▸), *SHELXT2018/2* (Sheldrick, 2015*a* ▸), *SHELXL2018/3* (Sheldrick, 2015*b* ▸) and *OLEX2* (Dolomanov *et al.*, 2009 ▸).

## full crystallographic data

### 2-Amino-5-oxo-4-(thiophen-2-yl)-5,6,7,8-tetrahydro-4H-chromene-3-carbonitrile Crystal data

**Table:**

C_14_H_12_N_2_O_2_S	*Z* = 4
*M**_r_* = 272.32	*F*(000) = 568
Triclinic, *P*1	*D*_x_ = 1.337 Mg m^−^^3^
*a* = 8.6822 (2) Å	Mo *K*α radiation, λ = 0.71073 Å
*b* = 10.0623 (2) Å	Cell parameters from 9904 reflections
*c* = 16.5504 (4) Å	θ = 2.3–27.2°
α = 77.201 (1)°	µ = 0.24 mm^−^^1^
β = 84.053 (1)°	*T* = 296 K
γ = 73.839 (1)°	Needle, yellow
*V* = 1352.89 (5) Å^3^	0.22 × 0.18 × 0.13 mm

### 2-Amino-5-oxo-4-(thiophen-2-yl)-5,6,7,8-tetrahydro-4H-chromene-3-carbonitrile Data collection

**Table:**

Bruker APEXII CCD diffractometer	5315 independent reflections
Radiation source: microfocus sealed X-ray tube, Incoatec Iµs	3991 reflections with *I* > 2σ(*I*)
Graphite monochromator	*R*_int_ = 0.030
Detector resolution: 7.9 pixels mm^-1^	θ_max_ = 26.0°, θ_min_ = 1.3°
φ and ω scans	*h* = −10→10
Absorption correction: multi-scan (SADABS; Krause *et al.*, 2015)	*k* = −12→12
*T*_min_ = 0.710, *T*_max_ = 0.746	*l* = −20→20
31629 measured reflections	

### 2-Amino-5-oxo-4-(thiophen-2-yl)-5,6,7,8-tetrahydro-4H-chromene-3-carbonitrile Refinement

**Table:**

Refinement on *F*^2^	Primary atom site location: dual
Least-squares matrix: full	Secondary atom site location: difference Fourier map
*R*[*F*^2^ > 2σ(*F*^2^)] = 0.044	Hydrogen site location: mixed
*wR*(*F*^2^) = 0.127	H atoms treated by a mixture of independent and constrained refinement
*S* = 1.05	*w* = 1/[σ^2^(*F*_o_^2^) + (0.0601*P*)^2^ + 0.4153*P*] where *P* = (*F*_o_^2^ + 2*F*_c_^2^)/3
5315 reflections	(Δ/σ)_max_ = 0.001
368 parameters	Δρ_max_ = 0.36 e Å^−^^3^
14 restraints	Δρ_min_ = −0.37 e Å^−^^3^

### 2-Amino-5-oxo-4-(thiophen-2-yl)-5,6,7,8-tetrahydro-4H-chromene-3-carbonitrile Fractional atomic coordinates and isotropic or equivalent isotropic displacement parameters (Å^2^)

**Table:**

	*x*	*y*	*z*	*U*_iso_*/*U*_eq_	Occ. (<1)
S2	0.10201 (8)	0.94938 (7)	0.60311 (4)	0.0711 (2)	
O3	0.42624 (18)	0.71195 (16)	0.48925 (8)	0.0573 (4)	
O4	0.49178 (17)	0.66307 (15)	0.77429 (8)	0.0510 (3)	
N3	0.2673 (3)	0.6174 (2)	0.43486 (12)	0.0666 (5)	
H3A	0.1778 (15)	0.597 (3)	0.4339 (16)	0.080*	
H3B	0.321 (3)	0.640 (3)	0.3895 (8)	0.080*	
N4	0.0220 (3)	0.4797 (2)	0.60077 (13)	0.0712 (6)	
C15	0.1180 (3)	0.5409 (2)	0.59319 (12)	0.0498 (5)	
C16	0.2381 (2)	0.6149 (2)	0.58188 (11)	0.0438 (4)	
C17	0.3050 (3)	0.6454 (2)	0.50498 (12)	0.0485 (5)	
C18	0.4997 (2)	0.7261 (2)	0.55589 (12)	0.0478 (5)	
C19	0.4398 (2)	0.70019 (19)	0.63378 (11)	0.0418 (4)	
C20	0.2793 (2)	0.66725 (19)	0.65438 (11)	0.0395 (4)	
H20	0.290103	0.590437	0.703329	0.047*	
C21	0.6484 (3)	0.7723 (3)	0.52901 (14)	0.0622 (6)	
H21A	0.630047	0.844621	0.478774	0.075*	
H21B	0.733930	0.692872	0.516795	0.075*	
C22	0.6987 (3)	0.8305 (3)	0.59647 (16)	0.0734 (7)	
H22A	0.806529	0.841406	0.583168	0.088*	
H22B	0.627145	0.922980	0.598604	0.088*	
C23	0.6942 (3)	0.7334 (3)	0.68045 (15)	0.0683 (7)	
H23A	0.778320	0.646518	0.680706	0.082*	
H23B	0.716365	0.778096	0.722603	0.082*	
C24	0.5369 (2)	0.6985 (2)	0.70210 (12)	0.0446 (4)	
C25	0.1501 (2)	0.7936 (2)	0.67445 (11)	0.0436 (4)	
C26	0.0545 (3)	0.8058 (3)	0.74616 (14)	0.0595 (5)	
H26	0.062218	0.734092	0.793156	0.071*	
C27	−0.0554 (3)	0.9393 (3)	0.7394 (2)	0.0844 (8)	
H27	−0.129952	0.964223	0.781700	0.101*	
C28	−0.0440 (3)	1.0265 (3)	0.6680 (2)	0.0856 (9)	
H28	−0.107778	1.118749	0.654771	0.103*	
O1	0.56105 (15)	0.28654 (14)	1.02275 (7)	0.0454 (3)	
O2	0.7051 (2)	0.3316 (2)	0.73827 (9)	0.0738 (5)	
N1	0.3186 (2)	0.3670 (2)	1.07922 (10)	0.0531 (4)	
H1A	0.2157 (3)	0.398 (2)	1.0812 (14)	0.064*	
H1B	0.372 (2)	0.354 (2)	1.1226 (8)	0.064*	
N2	0.0503 (2)	0.5075 (2)	0.90516 (11)	0.0595 (5)	
C1	0.1816 (2)	0.4476 (2)	0.91835 (11)	0.0432 (4)	
C2	0.3455 (2)	0.37569 (18)	0.93099 (11)	0.0382 (4)	
C3	0.4013 (2)	0.34601 (19)	1.00834 (11)	0.0389 (4)	
C4	0.6690 (2)	0.27370 (19)	0.95603 (11)	0.0401 (4)	
C5	0.6239 (2)	0.29850 (19)	0.87822 (11)	0.0400 (4)	
C6	0.4509 (2)	0.33547 (19)	0.85652 (10)	0.0384 (4)	
H6	0.432398	0.419030	0.811480	0.046*	
C7	0.7462 (3)	0.2988 (2)	0.80992 (13)	0.0515 (5)	
C8	0.9179 (3)	0.2652 (3)	0.83094 (15)	0.0685 (7)	
H8A	0.945730	0.352477	0.829798	0.082*	
H8B	0.985389	0.219461	0.789125	0.082*	
C9	0.9518 (3)	0.1697 (3)	0.91552 (16)	0.0704 (7)	
H9A	1.061586	0.158856	0.928494	0.084*	
H9B	0.940187	0.077074	0.914302	0.084*	
C10	0.8381 (2)	0.2298 (2)	0.98259 (13)	0.0529 (5)	
H10A	0.847670	0.159001	1.033546	0.064*	
H10B	0.866823	0.310630	0.993272	0.064*	
C11	0.4122 (2)	0.2179 (2)	0.82559 (11)	0.0415 (4)	
S1	0.43755 (12)	0.05115 (8)	0.88552 (5)	0.0659 (3)	0.837 (2)
C12	0.3525 (6)	0.2187 (5)	0.7519 (3)	0.0724 (13)	0.837 (2)
H12	0.326137	0.298965	0.710130	0.087*	0.837 (2)
C13	0.3351 (17)	0.0874 (8)	0.7456 (5)	0.0737 (13)	0.837 (2)
H13	0.299889	0.070523	0.698581	0.088*	0.837 (2)
C14	0.3736 (11)	−0.0099 (5)	0.8132 (5)	0.0655 (10)	0.837 (2)
H14	0.366076	−0.102135	0.819845	0.079*	0.837 (2)
S1A	0.3589 (11)	0.2484 (7)	0.7296 (4)	0.0659 (3)	0.163 (2)
C12A	0.409 (3)	0.0840 (17)	0.8574 (12)	0.0724 (13)	0.163 (2)
H12A	0.438376	0.041108	0.911508	0.087*	0.163 (2)
C13A	0.361 (7)	0.010 (3)	0.805 (2)	0.0737 (13)	0.163 (2)
H13A	0.361086	−0.084666	0.818619	0.088*	0.163 (2)
C14A	0.314 (10)	0.097 (4)	0.735 (3)	0.0737 (13)	0.163 (2)
H14A	0.264412	0.075658	0.693790	0.088*	0.163 (2)

### 2-Amino-5-oxo-4-(thiophen-2-yl)-5,6,7,8-tetrahydro-4H-chromene-3-carbonitrile Atomic displacement parameters (Å^2^)

**Table:**

	*U*^11^	*U*^22^	*U*^33^	*U*^12^	*U*^13^	*U*^23^
S2	0.0787 (4)	0.0567 (4)	0.0716 (4)	−0.0080 (3)	−0.0210 (3)	−0.0044 (3)
O3	0.0665 (9)	0.0756 (10)	0.0363 (7)	−0.0287 (8)	−0.0050 (6)	−0.0105 (7)
O4	0.0540 (8)	0.0610 (9)	0.0424 (8)	−0.0152 (7)	−0.0133 (6)	−0.0144 (6)
N3	0.0837 (15)	0.0846 (14)	0.0418 (10)	−0.0311 (12)	−0.0117 (10)	−0.0189 (10)
N4	0.0732 (13)	0.0932 (15)	0.0646 (12)	−0.0411 (12)	−0.0085 (10)	−0.0248 (11)
C15	0.0513 (12)	0.0588 (12)	0.0443 (11)	−0.0127 (10)	−0.0106 (9)	−0.0192 (9)
C16	0.0474 (11)	0.0473 (11)	0.0400 (10)	−0.0114 (9)	−0.0109 (8)	−0.0128 (8)
C17	0.0550 (12)	0.0521 (11)	0.0407 (10)	−0.0112 (10)	−0.0129 (9)	−0.0129 (9)
C18	0.0490 (11)	0.0523 (11)	0.0425 (10)	−0.0113 (9)	−0.0072 (9)	−0.0108 (9)
C19	0.0412 (10)	0.0449 (10)	0.0402 (10)	−0.0086 (8)	−0.0081 (8)	−0.0113 (8)
C20	0.0440 (10)	0.0433 (10)	0.0335 (9)	−0.0128 (8)	−0.0088 (7)	−0.0074 (7)
C21	0.0573 (13)	0.0757 (15)	0.0560 (13)	−0.0259 (12)	0.0033 (10)	−0.0100 (11)
C22	0.0640 (15)	0.0934 (19)	0.0765 (17)	−0.0420 (14)	0.0017 (13)	−0.0204 (14)
C23	0.0501 (13)	0.1007 (19)	0.0635 (14)	−0.0301 (13)	−0.0080 (11)	−0.0204 (13)
C24	0.0439 (11)	0.0447 (10)	0.0472 (11)	−0.0077 (9)	−0.0100 (9)	−0.0148 (8)
C25	0.0432 (10)	0.0497 (11)	0.0447 (10)	−0.0167 (9)	−0.0085 (8)	−0.0149 (8)
C26	0.0599 (13)	0.0687 (12)	0.0583 (13)	−0.0253 (9)	0.0061 (10)	−0.0236 (11)
C27	0.0637 (16)	0.0900 (17)	0.111 (2)	−0.0130 (12)	0.0103 (15)	−0.0593 (19)
C28	0.0677 (17)	0.0618 (16)	0.130 (3)	0.0017 (13)	−0.0277 (17)	−0.0390 (17)
O1	0.0371 (7)	0.0586 (8)	0.0357 (7)	−0.0034 (6)	−0.0062 (5)	−0.0093 (6)
O2	0.0749 (11)	0.1056 (14)	0.0436 (9)	−0.0294 (10)	0.0117 (8)	−0.0201 (8)
N1	0.0405 (9)	0.0784 (12)	0.0378 (9)	−0.0036 (9)	−0.0051 (7)	−0.0203 (8)
N2	0.0423 (10)	0.0775 (13)	0.0546 (11)	−0.0028 (9)	−0.0102 (8)	−0.0177 (9)
C1	0.0462 (12)	0.0486 (11)	0.0372 (10)	−0.0097 (9)	−0.0054 (8)	−0.0153 (8)
C2	0.0376 (10)	0.0385 (9)	0.0383 (9)	−0.0056 (8)	−0.0057 (7)	−0.0110 (7)
C3	0.0369 (10)	0.0410 (10)	0.0386 (9)	−0.0060 (8)	−0.0050 (7)	−0.0118 (8)
C4	0.0386 (10)	0.0396 (10)	0.0431 (10)	−0.0089 (8)	−0.0016 (8)	−0.0121 (8)
C5	0.0416 (10)	0.0392 (9)	0.0409 (10)	−0.0109 (8)	−0.0007 (8)	−0.0118 (8)
C6	0.0429 (10)	0.0388 (9)	0.0325 (9)	−0.0077 (8)	−0.0050 (7)	−0.0077 (7)
C7	0.0549 (12)	0.0567 (12)	0.0492 (12)	−0.0196 (10)	0.0082 (9)	−0.0222 (10)
C8	0.0513 (13)	0.0968 (19)	0.0706 (15)	−0.0288 (13)	0.0161 (11)	−0.0413 (14)
C9	0.0391 (12)	0.0877 (18)	0.0875 (18)	−0.0058 (12)	−0.0023 (11)	−0.0384 (15)
C10	0.0409 (11)	0.0601 (13)	0.0588 (12)	−0.0091 (9)	−0.0089 (9)	−0.0162 (10)
C11	0.0398 (10)	0.0481 (11)	0.0374 (9)	−0.0080 (8)	−0.0034 (8)	−0.0139 (8)
S1	0.0967 (7)	0.0480 (4)	0.0577 (5)	−0.0199 (4)	−0.0220 (4)	−0.0107 (3)
C12	0.087 (2)	0.065 (3)	0.068 (3)	−0.025 (2)	−0.034 (2)	0.0009 (19)
C13	0.081 (5)	0.096 (2)	0.065 (3)	−0.031 (3)	−0.018 (2)	−0.0414 (19)
C14	0.073 (3)	0.0532 (18)	0.077 (2)	−0.017 (2)	0.0004 (19)	−0.0270 (17)
S1A	0.0967 (7)	0.0480 (4)	0.0577 (5)	−0.0199 (4)	−0.0220 (4)	−0.0107 (3)
C12A	0.087 (2)	0.065 (3)	0.068 (3)	−0.025 (2)	−0.034 (2)	0.0009 (19)
C13A	0.081 (5)	0.096 (2)	0.065 (3)	−0.031 (3)	−0.018 (2)	−0.0414 (19)
C14A	0.081 (5)	0.096 (2)	0.065 (3)	−0.031 (3)	−0.018 (2)	−0.0414 (19)

### 2-Amino-5-oxo-4-(thiophen-2-yl)-5,6,7,8-tetrahydro-4H-chromene-3-carbonitrile Geometric parameters (Å, º)

**Table:**

S2—C25	1.716 (2)	N1—H1B	0.8600 (10)
S2—C28	1.704 (3)	N1—C3	1.337 (2)
O3—C17	1.373 (3)	N2—C1	1.149 (2)
O3—C18	1.382 (2)	C1—C2	1.419 (3)
O4—C24	1.225 (2)	C2—C3	1.357 (2)
N3—H3A	0.8600 (10)	C2—C6	1.517 (2)
N3—H3B	0.8600 (10)	C4—C5	1.335 (3)
N3—C17	1.344 (2)	C4—C10	1.493 (3)
N4—C15	1.149 (3)	C5—C6	1.505 (3)
C15—C16	1.416 (3)	C5—C7	1.468 (3)
C16—C17	1.351 (3)	C6—H6	0.9800
C16—C20	1.523 (2)	C6—C11	1.517 (3)
C18—C19	1.337 (3)	C7—C8	1.494 (3)
C18—C21	1.485 (3)	C8—H8A	0.9700
C19—C20	1.509 (3)	C8—H8B	0.9700
C19—C24	1.473 (3)	C8—C9	1.517 (4)
C20—H20	0.9800	C9—H9A	0.9700
C20—C25	1.517 (3)	C9—H9B	0.9700
C21—H21A	0.9700	C9—C10	1.520 (3)
C21—H21B	0.9700	C10—H10A	0.9700
C21—C22	1.521 (3)	C10—H10B	0.9700
C22—H22A	0.9700	C11—S1	1.717 (2)
C22—H22B	0.9700	C11—C12	1.371 (5)
C22—C23	1.515 (3)	C11—S1A	1.639 (6)
C23—H23A	0.9700	C11—C12A	1.340 (14)
C23—H23B	0.9700	S1—C14	1.664 (6)
C23—C24	1.493 (3)	C12—H12	0.9300
C25—C26	1.384 (3)	C12—C13	1.399 (7)
C26—H26	0.9300	C13—H13	0.9300
C26—C27	1.404 (4)	C13—C14	1.316 (5)
C27—H27	0.9300	C14—H14	0.9300
C27—C28	1.319 (4)	S1A—C14A	1.66 (2)
C28—H28	0.9300	C12A—H12A	0.9300
O1—C3	1.372 (2)	C12A—C13A	1.42 (2)
O1—C4	1.377 (2)	C13A—H13A	0.9300
O2—C7	1.221 (3)	C13A—C14A	1.315 (17)
N1—H1A	0.8600 (10)	C14A—H14A	0.9300
			
C28—S2—C25	92.21 (14)	C3—C2—C6	122.88 (16)
C17—O3—C18	118.29 (15)	N1—C3—O1	110.24 (15)
H3A—N3—H3B	121 (2)	N1—C3—C2	128.33 (17)
C17—N3—H3A	118.8 (18)	C2—C3—O1	121.42 (16)
C17—N3—H3B	118.9 (18)	O1—C4—C10	111.39 (15)
N4—C15—C16	178.5 (2)	C5—C4—O1	122.87 (16)
C15—C16—C20	119.63 (17)	C5—C4—C10	125.73 (17)
C17—C16—C15	118.39 (17)	C4—C5—C6	122.77 (16)
C17—C16—C20	121.82 (17)	C4—C5—C7	119.17 (18)
N3—C17—O3	110.55 (18)	C7—C5—C6	117.95 (16)
N3—C17—C16	127.3 (2)	C2—C6—H6	107.6
C16—C17—O3	122.10 (16)	C5—C6—C2	108.66 (14)
O3—C18—C21	111.46 (17)	C5—C6—H6	107.6
C19—C18—O3	122.36 (18)	C5—C6—C11	111.93 (15)
C19—C18—C21	126.18 (18)	C11—C6—C2	113.07 (15)
C18—C19—C20	122.39 (16)	C11—C6—H6	107.6
C18—C19—C24	118.72 (18)	O2—C7—C5	119.7 (2)
C24—C19—C20	118.84 (16)	O2—C7—C8	122.03 (19)
C16—C20—H20	108.2	C5—C7—C8	118.24 (19)
C19—C20—C16	108.00 (15)	C7—C8—H8A	109.1
C19—C20—H20	108.2	C7—C8—H8B	109.1
C19—C20—C25	111.81 (15)	C7—C8—C9	112.65 (19)
C25—C20—C16	112.35 (15)	H8A—C8—H8B	107.8
C25—C20—H20	108.2	C9—C8—H8A	109.1
C18—C21—H21A	109.5	C9—C8—H8B	109.1
C18—C21—H21B	109.5	C8—C9—H9A	109.4
C18—C21—C22	110.55 (18)	C8—C9—H9B	109.4
H21A—C21—H21B	108.1	C8—C9—C10	111.3 (2)
C22—C21—H21A	109.5	H9A—C9—H9B	108.0
C22—C21—H21B	109.5	C10—C9—H9A	109.4
C21—C22—H22A	109.4	C10—C9—H9B	109.4
C21—C22—H22B	109.4	C4—C10—C9	110.25 (17)
H22A—C22—H22B	108.0	C4—C10—H10A	109.6
C23—C22—C21	111.2 (2)	C4—C10—H10B	109.6
C23—C22—H22A	109.4	C9—C10—H10A	109.6
C23—C22—H22B	109.4	C9—C10—H10B	109.6
C22—C23—H23A	109.0	H10A—C10—H10B	108.1
C22—C23—H23B	109.0	C6—C11—S1	121.78 (13)
H23A—C23—H23B	107.8	C6—C11—S1A	118.6 (3)
C24—C23—C22	113.06 (19)	C12—C11—C6	130.4 (2)
C24—C23—H23A	109.0	C12—C11—S1	107.9 (2)
C24—C23—H23B	109.0	C12A—C11—C6	136.1 (8)
O4—C24—C19	120.29 (18)	C12A—C11—S1A	105.3 (8)
O4—C24—C23	121.58 (18)	C14—S1—C11	93.38 (18)
C19—C24—C23	118.05 (18)	C11—C12—H12	123.2
C20—C25—S2	120.92 (14)	C11—C12—C13	113.5 (4)
C26—C25—S2	109.92 (16)	C13—C12—H12	123.2
C26—C25—C20	129.15 (19)	C12—C13—H13	123.5
C25—C26—H26	124.1	C14—C13—C12	113.0 (4)
C25—C26—C27	111.9 (2)	C14—C13—H13	123.5
C27—C26—H26	124.1	S1—C14—H14	123.9
C26—C27—H27	122.8	C13—C14—S1	112.2 (4)
C28—C27—C26	114.3 (3)	C13—C14—H14	123.9
C28—C27—H27	122.8	C11—S1A—C14A	97.3 (12)
S2—C28—H28	124.2	C11—C12A—H12A	121.4
C27—C28—S2	111.7 (2)	C11—C12A—C13A	117.1 (15)
C27—C28—H28	124.2	C13A—C12A—H12A	121.4
C3—O1—C4	118.88 (13)	C12A—C13A—H13A	125.2
H1A—N1—H1B	120 (2)	C14A—C13A—C12A	110 (2)
C3—N1—H1A	121.5 (16)	C14A—C13A—H13A	125.2
C3—N1—H1B	117.9 (15)	S1A—C14A—H14A	125.1
N2—C1—C2	177.5 (2)	C13A—C14A—S1A	109.8 (19)
C1—C2—C6	117.98 (15)	C13A—C14A—H14A	125.1
C3—C2—C1	119.15 (16)		
			
S2—C25—C26—C27	1.3 (2)	C1—C2—C6—C5	164.50 (16)
O3—C18—C19—C20	−6.0 (3)	C1—C2—C6—C11	−70.6 (2)
O3—C18—C19—C24	171.55 (17)	C2—C6—C11—S1	−63.9 (2)
O3—C18—C21—C22	162.46 (19)	C2—C6—C11—C12	115.0 (4)
C15—C16—C17—O3	−177.49 (18)	C2—C6—C11—S1A	121.2 (4)
C15—C16—C17—N3	2.3 (3)	C2—C6—C11—C12A	−59.1 (16)
C15—C16—C20—C19	162.83 (17)	C3—O1—C4—C5	−9.4 (3)
C15—C16—C20—C25	−73.4 (2)	C3—O1—C4—C10	170.46 (16)
C16—C20—C25—S2	−62.0 (2)	C3—C2—C6—C5	−15.4 (2)
C16—C20—C25—C26	117.1 (2)	C3—C2—C6—C11	109.5 (2)
C17—O3—C18—C19	−11.9 (3)	C4—O1—C3—N1	−171.63 (16)
C17—O3—C18—C21	168.12 (18)	C4—O1—C3—C2	8.4 (3)
C17—C16—C20—C19	−21.9 (2)	C4—C5—C6—C2	14.6 (2)
C17—C16—C20—C25	101.9 (2)	C4—C5—C6—C11	−111.03 (19)
C18—O3—C17—N3	−168.63 (18)	C4—C5—C7—O2	−174.59 (19)
C18—O3—C17—C16	11.2 (3)	C4—C5—C7—C8	2.5 (3)
C18—C19—C20—C16	21.4 (2)	C5—C4—C10—C9	−18.9 (3)
C18—C19—C20—C25	−102.7 (2)	C5—C6—C11—S1	59.2 (2)
C18—C19—C24—O4	−173.63 (18)	C5—C6—C11—C12	−121.9 (4)
C18—C19—C24—C23	3.2 (3)	C5—C6—C11—S1A	−115.7 (4)
C18—C21—C22—C23	47.1 (3)	C5—C6—C11—C12A	64.1 (15)
C19—C18—C21—C22	−17.5 (3)	C5—C7—C8—C9	27.9 (3)
C19—C20—C25—S2	59.58 (19)	C6—C2—C3—O1	5.0 (3)
C19—C20—C25—C26	−121.3 (2)	C6—C2—C3—N1	−174.93 (19)
C20—C16—C17—O3	7.2 (3)	C6—C5—C7—O2	1.7 (3)
C20—C16—C17—N3	−173.0 (2)	C6—C5—C7—C8	178.74 (19)
C20—C19—C24—O4	4.1 (3)	C6—C11—S1—C14	179.9 (4)
C20—C19—C24—C23	−179.11 (19)	C6—C11—C12—C13	179.0 (7)
C20—C25—C26—C27	−177.9 (2)	C6—C11—S1A—C14A	−174 (3)
C21—C18—C19—C20	173.9 (2)	C6—C11—C12A—C13A	179 (3)
C21—C18—C19—C24	−8.5 (3)	C7—C5—C6—C2	−161.54 (16)
C21—C22—C23—C24	−53.0 (3)	C7—C5—C6—C11	72.9 (2)
C22—C23—C24—O4	−155.5 (2)	C7—C8—C9—C10	−53.4 (3)
C22—C23—C24—C19	27.7 (3)	C8—C9—C10—C4	48.0 (3)
C24—C19—C20—C16	−156.23 (16)	C10—C4—C5—C6	176.85 (18)
C24—C19—C20—C25	79.7 (2)	C10—C4—C5—C7	−7.1 (3)
C25—S2—C28—C27	0.0 (2)	C11—S1—C14—C13	0.6 (10)
C25—C26—C27—C28	−1.4 (3)	C11—C12—C13—C14	2.5 (14)
C26—C27—C28—S2	0.8 (3)	C11—S1A—C14A—C13A	−9 (7)
C28—S2—C25—C20	178.51 (16)	C11—C12A—C13A—C14A	−5 (7)
C28—S2—C25—C26	−0.78 (17)	S1—C11—C12—C13	−2.0 (8)
O1—C4—C5—C6	−3.3 (3)	C12—C11—S1—C14	0.8 (4)
O1—C4—C5—C7	172.76 (16)	C12—C13—C14—S1	−1.8 (15)
O1—C4—C10—C9	161.20 (18)	S1A—C11—C12A—C13A	−2 (3)
O2—C7—C8—C9	−155.1 (2)	C12A—C11—S1A—C14A	6 (3)
C1—C2—C3—O1	−174.91 (17)	C12A—C13A—C14A—S1A	9 (8)
C1—C2—C3—N1	5.1 (3)		

### 2-Amino-5-oxo-4-(thiophen-2-yl)-5,6,7,8-tetrahydro-4H-chromene-3-carbonitrile Hydrogen-bond geometry (Å, º)

**Table:**

*D*—H···*A*	*D*—H	H···*A*	*D*···*A*	*D*—H···*A*
N3—H3*A*···N4^i^	0.86 (1)	2.25 (1)	3.089 (3)	164 (2)
N3—H3*B*···O2^ii^	0.86 (1)	2.10 (2)	2.794 (2)	138 (2)
N1—H1*A*···N2^iii^	0.86 (1)	2.25 (1)	3.105 (2)	174 (2)
N1—H1*B*···O4^iv^	0.86 (1)	2.13 (1)	2.984 (2)	176 (2)

Symmetry codes: (i) −*x*, −*y*+1, −*z*+1; (ii) −*x*+1, −*y*+1, −*z*+1; (iii) −*x*, −*y*+1, −*z*+2; (iv) −*x*+1, −*y*+1, −*z*+2.
